# Association between total antioxidant capacity and mortality in ischemic stroke patients

**DOI:** 10.1186/s13613-016-0143-7

**Published:** 2016-04-23

**Authors:** Leonardo Lorente, María M. Martín, Antonia Pérez-Cejas, Pedro Abreu-González, Luis Ramos, Mónica Argueso, Juan J. Cáceres, Jordi Solé-Violán, Alejandro Jiménez

**Affiliations:** Intensive Care Unit, Hospital Universitario de Canarias, Ofra, s/n, 38320 La Laguna, Santa Cruz de Tenerife Spain; Intensive Care Unit, Hospital Universitario Nuestra Señora de Candelaria, Crta del Rosario s/n, 38010 Santa Cruz de Tenerife, Spain; Laboratory Department, Hospital Universitario de Canarias, Ofra, s/n, 38320 La Laguna, Santa Cruz de Tenerife Spain; Department of Physiology, Faculty of Medicine, University of the La Laguna, Santa Cruz de Tenerife, Spain; Intensive Care Unit, Hospital General La Palma, Buenavista de Arriba s/n, Breña Alta, 38713 La Palma, Spain; Intensive Care Unit, Hospital Clínico Universitario de Valencia, Avda. Blasco Ibáñez nº17-19, 46004 Valencia, Spain; Intensive Care Unit, Hospital Insular, Plaza Dr. Pasteur s/n, 35016 Las Palmas de Gran Canaria, Spain; Intensive Care Unit, Hospital Universitario Dr. Negrín, CIBERES, Barranco de la Ballena s/n, 35010 Las Palmas de Gran Canaria, Spain; Research Unit, Hospital Universitario de Canarias, Ofra, s/n, 38320 La Laguna, Santa Cruz de Tenerife Spain

**Keywords:** Total antioxidant capacity, Ischemic stroke, Cerebral infarction, Patients, Mortality

## Abstract

**Objective:**

Data on circulating total antioxidant capacity (TAC) levels in ischemic stroke patients compared with healthy controls are limited and provided conflicting findings. There are not data about the association between circulating TAC levels, peroxidation state and outcome in patients with severe ischemic stroke. The objective of this study was to examine the relationship of TAC with 30-day mortality after severe ischemic stroke.

**Methods:**

This multicenter study included 58 patients with coma (Glasgow Coma Scale < 9) following severe malignant middle cerebral artery infarction (MMCAI). We measured circulating levels of TAC and malondialdehyde (MDA, a biomarker of lipid peroxidation) on day 1 of severe MMCAI diagnosis. The study endpoint was 30-day mortality.

**Results:**

Non-survivors (*n* = 29) showed higher serum TAC levels (*p* < 0.001) and higher serum MDA levels (*p* = 0.004) than survivors (*n* = 29). Multiple binomial logistic regression analysis showed that serum TAC levels were associated with 30-day mortality, after controlling for Glasgow Coma Scale and age (odds ratio 1.92; 95 % confidence interval 1.201–3.072; *p* = 0.006). There was a correlation between serum TAC and MDA levels (rho = 0.35; *p* = 0.008).

**Conclusions:**

This single-center study in severe MMCAI patients found an association between higher serum TAC levels and 30-day mortality and further identified a relationship between serum TAC levels, lipid peroxidation state and mortality after severe ischemic stroke.

## Background

Ischemic stroke leads to substantial disability, mortality and consumption of resources [1]. Increased reactive oxygen species (ROS) appears during ischemic stroke and are involved in cellular damage [2–8]. Antioxidant state may eventually be overwhelmed leading to oxidative stress, thereby exacerbating damage to proteins, lipids, carbohydrates and nucleic acids [2–8]. The different antioxidant agents act synergistically to cause further damage; thus, the determination of total antioxidant capacity (TAC) in serum or plasma could give more information about the antioxidant status than the determination of individual antioxidant compounds [9].

Circulating TAC in ischemic stroke patients has been scarcely studied, and the findings are conflicting [10–15]. In some studies, it was found that circulating TAC in ischemic stroke patients was lower than in healthy control subjects [10–13], while in another study no differences in circulating TAC between ischemic stroke patients and healthy control subjects were found [14]. Finally, in another study was found higher circulating TAC in ischemic stroke patients than in healthy control subjects [15]. However, there are not data about the association between circulating TAC levels, peroxidation state and mortality in patients with ischemic stroke. Thus, the objective of this study was to determine whether there is an association between circulating TAC levels, peroxidation state and mortality in patients with severe malignant middle cerebral artery infarction (MMCAI).

## Methods

### Design and patients

This multicenter, observational, prospective study included patients with severe malignant middle cerebral artery infarction (MMCAI). We used Glasgow Coma Scale (GCS) to assess the severity of MMCAI [16]. Comatose patients defined by a GCS lower than 9 were included. Patients with age less than 18 years, pregnancy, malignant or inflammatory disease were excluded.

The study was carried out in six Spanish intensive care units and was approved by the institutional review board of each participant hospitals: Hospital Universitario Nuestra Señora de Candelaria (Santa Cruz de Tenerife, Spain), Hospital Universitario de Canarias (La Laguna, Santa Cruz de Tenerife, Spain), Hospital Clínico Universitario de Valencia (Valencia, Spain), Hospital General de La Palma (La Palma, Spain), Hospital Universitario Dr. Negrín (Las Palmas de Gran Canaria, Spain) and Hospital Insular (Las Palmas de Gran Canaria, Spain). In addition, the written informed consent was obtained from legal representatives of the patients.

Previously, we determined serum malondialdehyde (MDA) levels (to assess the level of lipid peroxidation) in that cohort of patients with severe MMCAI [17]. In the current analysis, we have examined the association of serum TAC levels with mortality.

### Variables recorded

For each patient, the following variables were recorded: activated partial thromboplastin time (aPTT), age, Acute Physiology and Chronic Health Evaluation II (APACHE II) score [18], bilirubin, creatinine, decompressive craniectomy, fibrinogen, GCS, glycemia, hemoglobin, international normalized ratio (INR), lactic acid, leukocytes, pressure of arterial oxygen (PaO_2_), fraction inspired oxygen (FiO_2_) ratio, platelets, sex, sodium and temperature. The endpoint of the study was 30-day mortality.

### Blood sample collection

We collected blood samples in tubes with separator gel on day 1 of severe MMCAI diagnosis. We considered day 1 as the day that patients showed GCS < 9. Blood samples were obtained within the first 4 h of severe MMCAI diagnosis. We obtained serum samples by centrifugation at 1000×*g* for 15 min after coagulation during 10 min at room temperature. Then, the serum samples were aliquoted and frozen, until determination, at −80 °C.

### Determination of serum TAC levels

We determined TAC in serum samples using antioxidant assay kit (Cayman Chemical Corporation, Ann Arbor, USA). The assay is based on the ability to inhibit the oxidation of ABTS^®^ (2,2′-azino-di-[3-ethylbenzthiazoline sulfonate]) to ABTS^®+^ by metmyoglobin by antioxidants of the serum sample. The antioxidant capacity from the serum sample to prevent ABTS oxidation is compared with that by Trolox (a water-soluble tocopherol analog) and was quantified as molar Trolox equivalents. We assayed all samples, following manufacturer’s instructions, in duplicate at 20-fold dilutions in assay buffer. Absorbances at 750 nm using the EnSpire multimode plate reader (PerkinElmer, Waltham, MA, USA) were measured. Serum TAC levels were expressed in mmol/L. The assay detection limit was of 0.04 mmol/L; and the inter- and intra-assay coefficient of variation (CV) was 3.0 and 3.4 %, respectively. All serum samples were processed at the same time, at the end of the recruitment process, to avoid the possible dispersion of results. A laboratory technician blinded to all clinical data of the Laboratory Deparment of the Hospital Universitario de Canarias (La Laguna, Tenerife, Spain) processed all the samples.

### Determination of serum malondialdehyde (MDA) levels

To asses lipid peroxidation, serum MDA levels were measured [19]. MDA is an end product formed during degradation of cellular membrane phospholipids due to lipid peroxidation, after is released into extracellular space and the blood.

We determined serum MDA levels by thiobarbituric acid-reactive substance (TBARS) method described by Kikugawa et al. [20]. We use *n*-butanol to remove the pink complex samples. We placed each sample in a 96-well plate and read it in a microplate spectrophotometer reader (Benchmark Plus, Bio-Rad, Hercules, CA, USA) at 535 nm. The assay detection limit was of 0.079 nmol/ml. The inter- and intra-assay CV was 4.01 and 1.82 %, respectively. Serum MDA concentrations were expressed in nmol/ml. All serum samples were processed at the same time, at the end of the recruitment process, to avoid the possible dispersion of results. A laboratory technician blinded to all clinical data of the Department of Physiology, Faculty of Medicine (University of the La Laguna, Santa Cruz de Tenerife, Spain), processed all the samples.

### Statistical methods

We reported categorical variables as frequencies and percentages, and we carried out comparisons between groups using Chi-square test. We reported continuous variables as medians and interquartile ranges, and we carried out comparisons between groups using Wilcoxon–Mann–Whitney test.

Goodness of fit for serum TAC levels to predict 30-day mortality was carried out with a receiver operating characteristic (ROC) analysis. We performed a Kaplan–Meier of survival analysis, using serum TAC lower/higher than 3.39 mmol/mL as the independent variable and survival at 30 days as the dependent variable; and both curves were compared by log-rank test. We used Youden’s index to select the cutoff point for serum TAC levels.

We used multiple logistic regression analysis to determine the association between serum TAC levels and 30-day mortality, controlling for GCS and age. We calculated odds ratio and 95 % confidence intervals to measure the clinical impact of the predictor variables. The association between continuous variables was carried out using Spearman’s rank correlation test.

We performed statistical analyses using SPSS 17.0 (SPSS Inc., Chicago, IL, USA), NCSS 2000 (Kaysville, Utah) and LogXact 4.1, (Cytel Co., Cambridge, MA). All *p* values less than 0.05 were considered statistically significant.

## Results

**Fig. 1:**
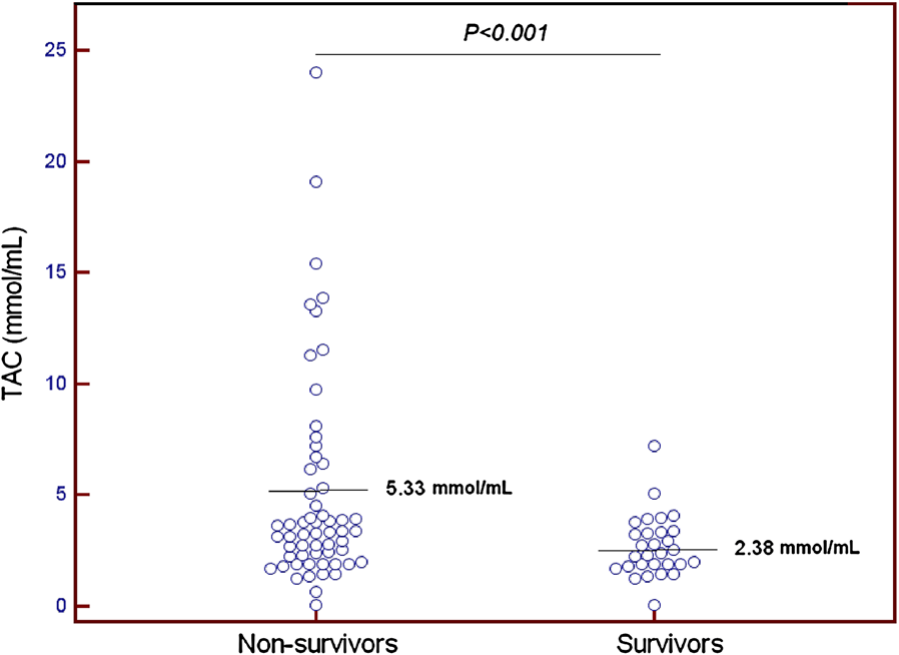
Dot plot of serum total antioxidant capacity (TAC) levels in survivors and non-survivors at 30 days

**Fig. 2:**
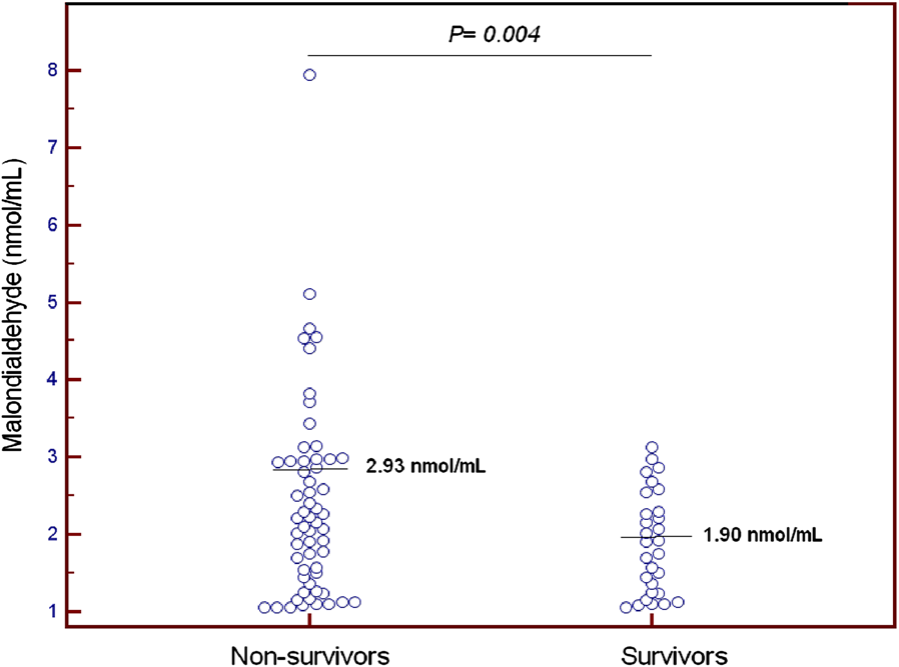
Dot plot of serum malondialdehyde levels in survivors and non-survivors at 30 days

Table 1 shows the comparisons between survivors (*N* = 29) and non-survivors (*N* = 29) MMCAI patients. Non-survivors had lower GCS (*p* = 0.02), higher serum TAC levels (*p* < 0.001) and higher serum MDA levels (*p* = 0.004) than survivors (Figs. 1, 2). No patient underwent endovascular thrombectomy.

**Table 1:** Comparison between survivor and non-survivor severe MMCAI patients

	Survivors (*n* = 29)	Non-survivors (n = 29)	*p* value
Sex female—*n* (%)	13 (44.8)	11 (37.9)	0.79
Age (years)—median (*p* 25–75)	57 (47–67)	64 (54–70)	0.08
Temperature (°C)—median (*p* 25–75)	36.4 (35.8–37.0)	37.0 (36.0–37.6)	0.18
C-reactive protein (mg/L)—median (*p* 25–75)	19 (5–45)	27 (12–48)	0.39
Timing of coma—*n* (%)			0.97
At hospital admission	19 (65.5)	20 (69.0)	
Within first 24 h of hospital admission	5 (17.2)	5 (17.2)	
24–48 h of hospital admission	1 (3.4)	1 (3.4)	
48–96 h of hospital admission	2 (6.9)	2 (6.9)	
More than 96 h of hospital admission	2 (6.9)	1 (3.4)	
Thrombolysis—*n* (%)	10 (34.5)	9 (31.0)	0.99
Decompressive craniectomy—*n* (%)	8 (27.6)	5 (17.2)	0.53
Sodium (mEq/L)—median (*p* 25–75)	139 (136–145)	140 (139–146)	0.41
Glycemia (g/dL)—median (*p* 25–75)	128 (100–170)	135 (105–160)	0.99
Leukocytes × 10^3^/mm^3^—median (*p* 25–75)	12.5 (9.5–16.9)	13.9 (9.3–21.4)	0.43
PaO_2_ (mmHg)—median (*p* 25–75)	137 (104–207)	114 (86–153)	0.26
PaO_2_/FiO_2_ ratio—median (*p* 25–75)	300 (197–372)	248 (184–330)	0.23
Bilirubin (mg/dl)—median (*p* 25–75)	0.70 (0.40–0.95)	0.70 (0.33–1.10)	0.87
Creatinine (mg/dl)—median (*p* 25–75)	0.80 (0.60–1.15)	1.00 (0.76–1.28)	0.12
Hemoglobin (g/dL)—median (*p* 25–75)	12.2 (11.4–14.4)	13.7 (11.0–14.9)	0.78
Glasgow Coma Scale score—median (*p* 25–75)	7 (6–8)	6 (3–7)	0.02
Lactic acid (mmol/L)—median (*p* 25–75)	1.30 (0.90–1.70)	1.40 (1.00–2.10)	0.25
Platelets × 10^3^/mm^3^—median (*p* 25–75)	214 (170–280)	170 (131–212)	0.008
INR—median (*p* 25–75)	1.09 (1.01–1.20)	1.20 (1.05–1.31)	0.10
aPTT (seconds)—median (*p* 25–75)	28 (26–30)	27 (26–32)	0.77
Fibrinogen (mg/dl)—median (*p* 25–75)	440 (335–494)	419 (311–631)	0.82
APACHE II score—median (*p* 25–75)	20 (16–26)	22 (19–28)	0.22
Malondialdehyde (nmol/mL)—median (*p* 25–75)	1.90 (1.24–2.42)	2.93 (1.83–3.77)	0.004
TAC (mmol/mL)—median (*p* 25–75)	2.38 (1.83–3.35)	5.33 (3.27–11.40)	<0.001

*p* percentile, *PaO*_*2*_ pressure of arterial oxygen, *FIO*_*2*_ pressure of arterial oxygen/fraction inspired oxygen, *INR* international normalized ratio, *aPTT* activated partial thromboplastin time, *APACHE II* Acute Physiology and Chronic Health Evaluation II, *TAC* total antioxidant capacity

**Fig. 3:**
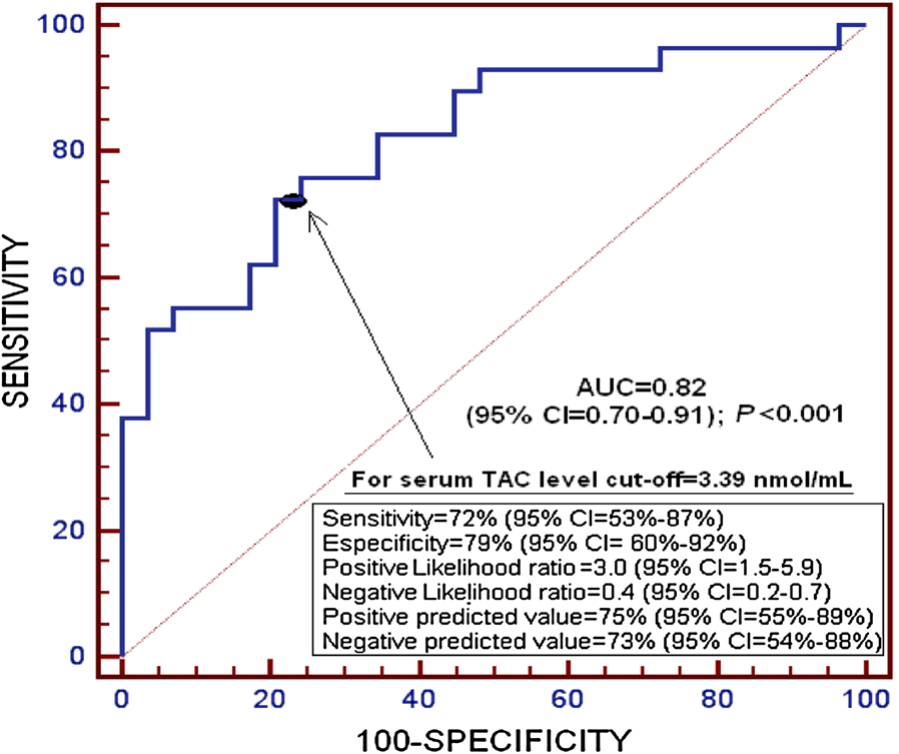
Receiver operation characteristic analysis using serum total antioxidant capacity (TAC) as predictor of mortality at 30 days

We found that the area under the curve (AUC) for serum TAC levels as predictor of 30-day mortality was of 0.82 (95 % CI 0.70–0.91; *p* < 0.001) (Fig. 3).

**Fig. 4:**
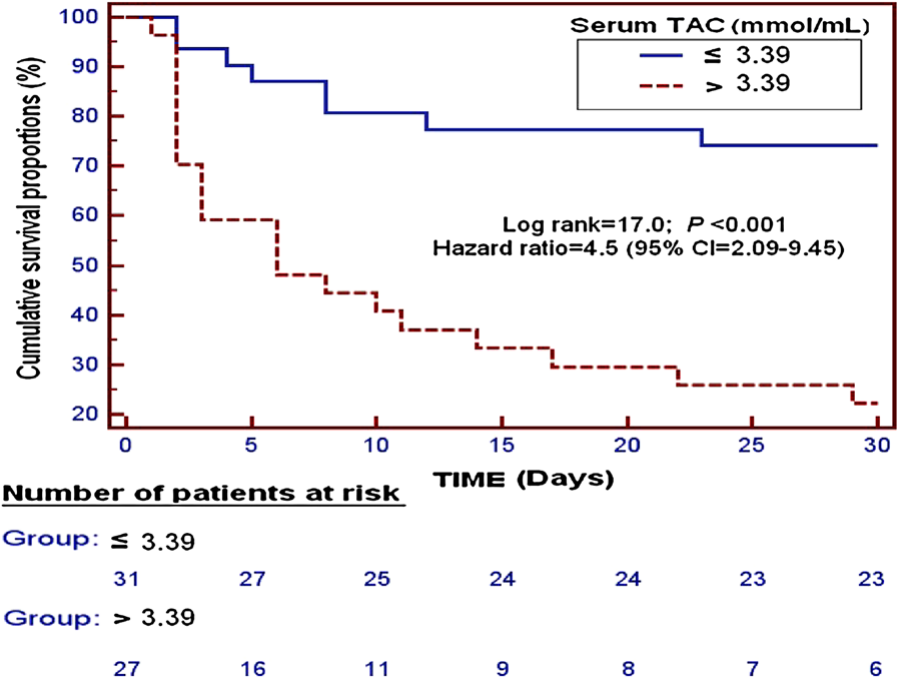
Survival curve at 30 days using 3.39 nmol/mL of serum total antioxidant capacity (TAC) as cutoff

In the survival analysis, patients with serum TAC higher than 3.39 mmol/mL had higher 30-day mortality than patients with lower serum TAC levels (hazard ratio 4.5; 95 % CI 2.09–9.45; *p* < 0.001) (Fig. 4).

We found in the multiple binomial logistic regression analysis that serum TAC levels were associated with 30-day mortality (odds ratio 1.92; 95 % CI 1.201–3.072; *p* = 0.006) after adjusting for GCS and age (Table 2).

**Table 2:** Multiple binomial logistic regression analysis to predict 30-day mortality

Variable	Odds ratio	95 % confidence interval	*p*
Glasgow Coma Scale (points)	0.69	0.478–0.997	0.048
Age (years)	1.02	0.964–1.077	0.51
Serum TAC levels (mmol/mL)	1.92	1.201–3.072	0.006

We also found a correlation between serum TAC and MDA levels (rho = 0.35; *p* = 0.008).

## Discussion

The main finding of our study was that in severe MMCAI patients non-survivors had higher serum TAC levels than survivors and that serum TAC levels could be used as a prognostic biomarker of mortality in MMCAI patients. We also found an association between serum TAC levels and lipid peroxidation state in severe MMCAI patients. 

Previously, some studies compared circulating TAC between ischemic stroke patients and healthy subjects, and there were found conflicting findings [10–15]. In our study, we found higher serum TAC levels in non-surviving than in surviving severe MMCAI patients for the first time. In addition, according to the results of the multiple logistic regression analysis, there was an association between circulating TAC levels and mortality in patients with severe MMCAI; and that may be the major novel finding of our study.

Some studies reported lower circulating TAC and higher circulating MDA levels in ischemic stroke patients than in healthy control subjects [10, 13]. However, in one study higher circulating TAC and nitric oxide levels in ischemic stroke patients than in healthy control subjects were found [15]. Furthermore, higher circulating hydroperoxides and a tendency to higher circulating TAC levels in ischemic stroke patients compared with healthy control subjects were observed [14]. The findings of our study are in line with some of those previous studies [14, 15]. However, while previous studies compared circulating TAC levels between ischemic stroke patients and healthy control subjects, in the present analysis we compared circulating TAC levels between non-survivors and survivor patients with severe ischemic stroke.

In addition, the findings of our study are in line with the results of other studies showing higher circulating TAC levels in non-survivor compared with survivor patients, and an association between circulating TAC levels and patient mortality, such as in septic patients [21] and traumatic brain injury patients [22]. We report for the first time that serum TAC levels could be used as a predictor biomarker of mortality in patients with severe MMCAI according to the results of ROC curve analysis. In addition, the other novel aspect of our study was the positive association between serum TAC levels and lipid peroxidation (assessed by serum MDA levels).

The association that we have found between increased serum TAC levels and mortality in patients with severe MMCAI could seem initially counterintuitive because improved protection against oxidative processes would instead protect the brain against reactive oxygen species (ROS)-induced damage. However, we think that the findings of previous studies showing a higher oxidative state in patients with ischemic stroke compared with healthy control subjects [10, 13–15], and the findings of our study showing higher circulating TAC and MDA levels in non-survivors compared to survivors patients with severe MMCAI could reflect that there is an oxidative state in stroke and that it is higher in non-surviving patients. We postulate that non-surviving MMCAI patients display higher serum TAC levels to compensate the higher ROS production, as it was observed by our group in severe septic patients [21] and in patients with severe traumatic brain injury [22]; thus, non-surviving patients showed higher serum levels of TAC and MDA than surviving patients.

From a therapeutic perspective, the development of antioxidant agents could be used as a new class of drugs for the treatment of patients with MMCAI. The use of melatonin has reduced circulating MDA levels and mortality rate in asphyxiated newborns patients [23] and septic newborns patients [24]. The administration of different antioxidant agents (vitamins E and C, zinc sulfate, allopurinol, melatonin and *N*-acetylcysteine) has reduced circulating MDA levels and mortality rate in burn patients [25]. The use of amantadine sulfate has reduced MDA levels and mortality in traumatic brain injury patients [26]. The administration of different antioxidant vitamins (E, C, B2, B6, B9 and B12) in acute ischemic stroke patients has been associated with a reduction in circulating MDA levels [27].

Some limitations should be recognized in our study. First, we did not determine serum TAC levels in healthy control subjects; however, the objective of our study was not to determine whether there is difference between ischemic stroke patients and healthy control subjects, but rather the association between serum TAC levels and mortality. Second, we did not measure serum TAC levels in patients with middle cerebral artery infarction without malignant criteria. Third, we did not determine serum TAC levels during follow-up. Fourth, we have found a higher variability (4.3 %) in serum TAC levels that mentioned by the manufacturer (3.4 %); however, our variability is acceptable. Fifth, the sensitivity and specificity of serum TAC levels as prognostic biomarker of mortality were not very high; however, both were statistically significant. We think that the sole use of serum TAC levels as prognostic biomarker of mortality in patients with severe malignant MMCAI should be taken with caution. Sixth, we did not report the National Institutes of Health Stroke Scale (NIHSS) due to the difficulty to evaluate the different items of this scale in our patients with GCS < 9. Seventh, the rate of decompressive craniectomy in our series was relatively low (22.4 %), although in line with published series [28–30]. Pending further larger studies, we believe that the findings of our study could open the way for future research on TAC and oxidative stress in ischemic stroke patients.

## Conclusions

Higher serum TAC levels are associated with mortality in patients with severe ischemic stroke and could be used as a prognostic biomarker of outcome.
